# Elevated *ex vivo *monocyte chemotactic protein-1 (CCL2) in pulmonary as compared with extra-pulmonary tuberculosis

**DOI:** 10.1186/1471-2172-6-14

**Published:** 2005-07-07

**Authors:** Zahra Hasan, Irfan Zaidi, Bushra Jamil, M Aslam Khan, Akbar Kanji, Rabia Hussain

**Affiliations:** 1 Department of Pathology and Microbiology, The Aga Khan University, Karachi, Pakistan; 2 Department of Medicine, The Aga Khan University, Karachi, Pakistan

## Abstract

**Background:**

Tuberculosis causes 3 million deaths annually. The most common site of tuberculosis is pulmonary however; extra-pulmonary forms of the disease also remain prevalent. Restriction of *Mycobacterium tuberculosis *depends on effective recruitment and subsequent activation of T lymphocytes, mononuclear and polymorphonuclear cells to the site of infection. Tumor necrosis factor (TNF)-α is essential for granuloma formation and is a potent activator of monocyte chemotactic protein (MCP-1, CCL2). CCL2 is essential for recruitment of monocytes and T cells and has been shown to play a role in protection against tuberculosis. Interleukin -8 (CXCL8) is a potent activator of neutrophils. Increased levels of CCL2, CXCL8 and TNFα are reported in tuberculosis but their significance in different forms of tuberculosis is as yet unclear. We have used an *ex vivo *assay to investigate differences in immune parameters in patients with either pulmonary or extra-pulmonary tuberculosis.

**Methods:**

Serum levels of CCL2, CXCL8 and TNFα were measured in patients with pulmonary tuberculosis (N = 12), extra-pulmonary tuberculosis (N = 8) and BCG-vaccinated healthy volunteers (N = 12). Whole blood cells were stimulated with non-pathogenic *Mycobacterium bovis *bacille-Calmette Guerin (BCG) vaccine strain or bacterial lipopolysaccharide (LPS) and cyto/chemokines were monitored in supernatants.

**Results:**

Circulating serum levels of CXCL8 and TNFα were raised in all tuberculosis patients, while CCL2 levels were not. There was no difference in spontaneous cytokine secretion from whole blood cells between patients and controls. *M. bovis *BCG-induced *ex vivo *CCL2 secretion was significantly greater in pulmonary as compared with both extra-pulmonary tuberculosis patients and endemic controls. In response to LPS stimulation, patients with pulmonary tuberculosis showed increased CCL2 and TNFα responses as compared with the extra-pulmonary group. BCG-, and LPS-induced CXCL8 secretion was comparable between patients and controls.

**Conclusion:**

CCL2 is activated by TNFα and is essential for recruitment of monocytes and T cells to the site of mycobacterial infection. Increased CCL2 activation in pulmonary tuberculosis may result in a stronger cellular response as compared with extra-pulmonary tuberculosis patients, and this may contribute to the localization of infection to the pulmonary site.

## Background

Tuberculosis is a major cause of morbidity and mortality worldwide, causing approximately 3 millions deaths annually [1]. *Mycobacterium tuberculosis*, the causative agent of tuberculosis is a successful pathogen due to its ability to down regulate host immune responses. The primary site of infection for *M. tuberculosis *is the alveolar macrophage. In cases where pulmonary infection cannot be controlled the organism disseminates via blood to other sites. Pulmonary involvement is seen in the majority of tuberculosis cases however infections of extra-pulmonary sites such as lymph nodes, skeletal, abdominal and genito-urinary sites also remain common [2].

Local cellular responses required for restriction of infection with *M. tuberculosis *are fairly well understood. Granulomas which are the characteristic histopathologic lesions of tuberculosis can be formed in any infected tissue. Granulomas exhibit a characteristic mixture of macrophages and lymphocytes and the outcome of infection depends on the interplay between immune activating cytokines produced at this site. T cell interferon -gamma (IFN-γ) and macrophage activating tumor necrosis factor-alpha (TNFα) are critical for protection and play a central role in granuloma formation [3,4]. Recent studies have also indicated an important role for chemokines in granuloma formation [5,6].

Chemokines are small mass chemotactic cytokines produced by epithelial cells, mast cells, monocytes and neutrophils. Chemokines can be grouped into structurally different families including C-C chemokines; monocyte chemotactic protein-1 (MCP-1, CCL2), macrophage inflammatory protein-1α (MIP-1α, CCL3), MIP-1β (CCL4) and regulated-upon-activation, normally T-cell-expressed and-secreted (RANTES, CCL5) and C-X-C chemokines such as, IL-8 (CXCL8), MIG (CXCL9) and IP-10 (CXCL10). These are all activated by *M. tuberculosis *[7]. CCL2 is the most potent chemoattractant and activator for monocytes and attracts CD4 and γδ T cells [8]. CCL2 has also been shown to play a role in protection against murine tuberculosis [9-11]. CXCL8 is the most potent attractant and activating factor for neutrophils and is chemotactic for lymphocytes [12,13]. CXCL8 is required for stimulation of a pro-inflammatory response against *M. tuberculosis *and its components [14].

Previous studies have shown that *in vivo *(circulating) serum levels of IFNγ, IL-10 [15], TNFα [16], CXCL8 and IL-6 [17] are raised in pulmonary tuberculosis. Circulating levels of chemokines CXCL8, CCL2 and RANTES are raised in bronchoalveolar lavage fluid (BALF) and alveolar macrophages from pulmonary tuberculosis patients [18,19]. Increased CXCL8 and CCL2 activation is also observed in patients with tuberculous pleurisy [20] and meningitis [21,22]. In addition, the expression levels of cytokines and chemokines have been correlated with disease severity [15] and with subsequent recovery after treatment [23,24].

We have focused on immune responses in newly diagnosed untreated tuberculosis patients with either pulmonary, or extra-pulmonary disease. We observed that systemic levels of CXCL8 and TNFα were raised in all patients while CCL2 was not. Using an *ex vivo *whole blood assay we observed differential *Mycobacterium*-induced and LPS stimulated responses between patients with pulmonary and extra-pulmonary tuberculosis. *Mycobacterium*-induced CCL2 and TNFα, and LPS-induced TNFα responses were greater in pulmonary as compared with extra-pulmonary tuberculosis. However, both *Mycobacterium*-, and LPS-induced CXCL8 responses were comparable between patient groups.

## Results

### Patient characteristics

Hematological characteristics of tuberculosis patients and healthy endemic controls are shown in Table 1. Median values obtained for pulmonary (Pul-TB) and extra-pulmonary tuberculosis (EPul-TB) were not different from controls, although for total lymphocyte and neutrophil counts were higher in tuberculosis patients. The erythrocyte sedimentation rate was raised in both patient groups as compared with controls.

### Serum CCL2, CXCL8 and TNFα in tuberculosis patients

**Figure 1:**
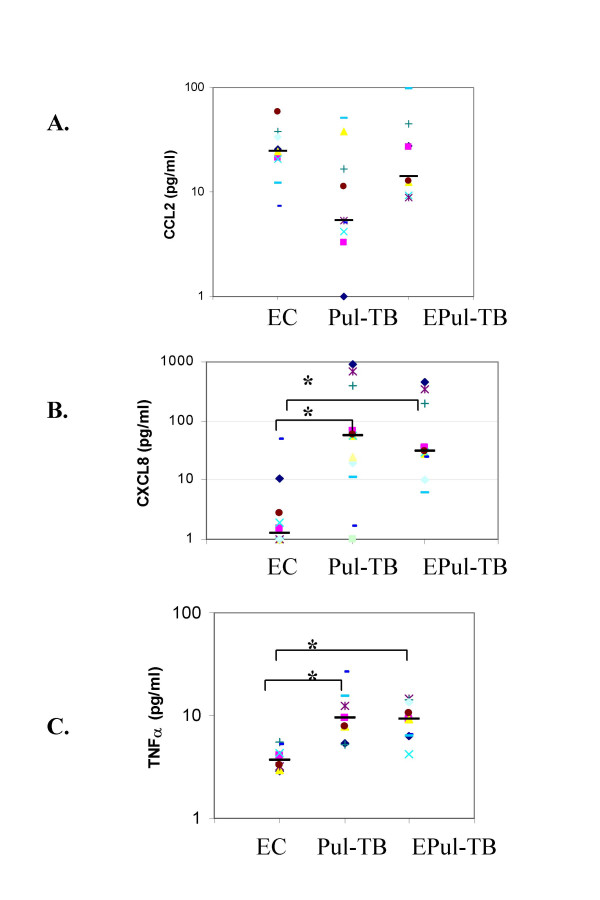
**Raised CXCL8 and TNFα in sera of tuberculosis patients. **Circulating levels of chemo/cytokines were determined in sera of endemic controls (EC, N = 12), pulmonary tuberculosis (Pul-TB, N = 10) and extra-pulmonary tuberculosis (EPul-TB, N = 8) patients. Values for individual subjects are shown, with the median value indicated by a horizontal line. A. CCL2, B. CXCL8 and C. TNFα. Statistical analysis was carried out using the Mann Whitney-U test with significant differences, P < 0.05, '*'.

We determined circulating levels of CCL2, CXCL8 and TNFα in sera of pulmonary and extra-pulmonary tuberculosis patients and controls (Figure 1). No difference was observed in serum CCL2 levels between the Pul-TB, EPul-TB and control (EC) groups (Fig. 1A). However, serum CXCL8 was significantly greater in both Pul-TB (P = 0.003) and EPul-TB (P = 0.002) as compared with controls (Fig. 1B). Serum TNFα was also significantly greater in both Pul-TB (P = 0.001) and in EPul-TB (P = 0.001) as compared with controls (Fig. 1C).

### BCG-, and LPS -induced CCL2, CXCL8 and TNFα

**Figure 2:**
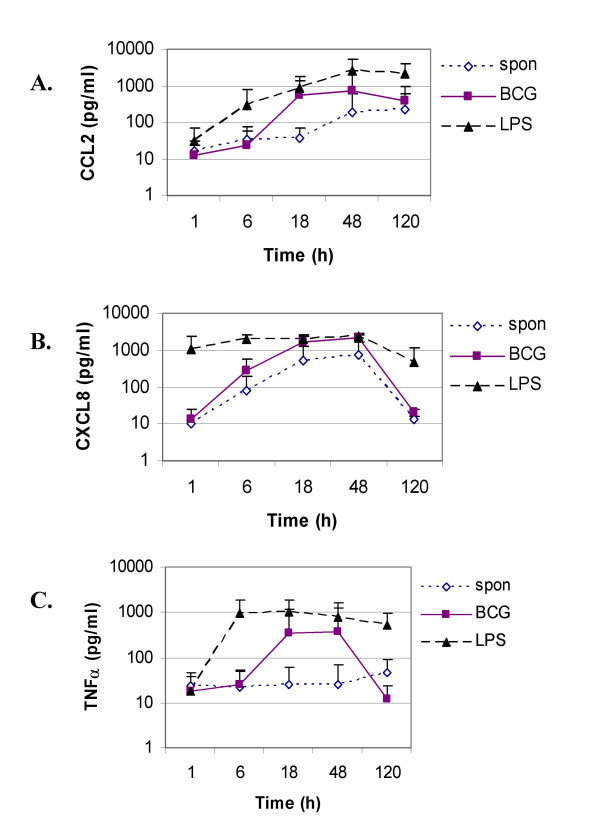
**Time-dependent secretion of *Mycobacterium*-, and LPS- induced CCL2, CXCL8 and TNFα. **Whole blood cells (10^6^) from healthy controls (N = 12) were stimulated with *M. bovis *BCG (2 × 10^5 ^CFU/ml, ratio of 0.2 bacilli per cell) and LPS at 0.1 μg/ml. Supernatants were harvested at 1, 6, 18, 48 and 120 h post-stimulation and tested for cytokine activity. Mean values are illustrated with SD indicated as 'y' error bars. A. CCL2, B. CXCL8, C. TNFα. Un-stimulated cells (spon) '◇', BCG stimulated '■' and LPS stimulated '▲'.

A time course profile of *M. bovis *BCG – and LPS-induced CCL2, CXCL8 and TNFα secretion was determined in healthy controls, see Fig. 2. BCG infection induced CXCL8 secretion within 6 – 18 h post stimulation (Fig. 2B), followed by TNFα (Fig. 2C) and then by CCL2 (Fig. 2A). LPS induced higher levels of all three cytokines as compared with BCG. In addition, CXCL8, TNF α and CCL2 were secreted more rapidly by LPS. Peak secretion of chemo/cytokines was observed between 18 and 48 h post-stimulation, therefore BCG and LPS induced responses were subsequently studied at these time intervals.

### CCL2, CXCL8 and TNFα secretion in whole blood cells from tuberculosis patients

Baseline (spontaneous) levels of CCL2, CXCL8 and TNFα were determined in Pul-TB, EPul-TB and control groups. Chemokine activity was measured in supernatants of un-stimulated whole blood cells after 18 h and 48 h of *ex vivo *culture. At 18 h, increased levels of CCL2 were observed in the Pul-TB group; 438 pg/ml (median) (range, 0–3000 pg/ml) as compared with EPul-TB; 35 pg/ml (0–774 pg/ml) and controls; 42 pg/ml (0–102 pg/ml). Spontaneous secretion of CXCL8 after 18 h was : Pul-TB; 284 pg/ml (15–1927 pg/ml), EPul-TB; 681 pg/ml (1–2076 pg/ml) and controls; 121 pg/ml (8–2546 pg/ml). Spontaneous TNFα secretion at 18 h was: Pul-TB; 5 pg/ml (0–55 pg/ml), E-Pul-TB; 1 pg/ml (0–585 pg/ml) and controls; 25 pg/ml (0–102 pg/ml). There were no significant differences between groups in their baseline levels of CCL2, CXCL8 or TNFα (P > 0.05). Spontaneous levels of cytokines measured after 48 h (data not shown) of *in vitro *culture showed a similar trend as observed at 18 h, with no difference between groups.

We subsequently investigated LPS-induced CCL2, CXCL8 and TNFα in tuberculosis patients. Table 2 illustrates chemokine activity measured at 18 and 48 h post LPS stimulation. Spontaneous chemokine secretion (indicated above) have been subtracted from the values indicated in the table. LPS-induced CCL2 activation in Pul-TB was significantly greater than in EPul-TB at 18 h (*^1^, P = 0.01) and at 48 h (*^2^, P = 0.02) post-stimulation. LPS-induced CXCL8 secretion was comparable between patients and endemic controls. At 18 h, TNFα was significantly greater in Pul-TB as compared with EPul-TB (*^4^, P = 0.003) and also as compared with controls (*^3^, P = 0.003), whilst no difference was observed between these groups at 48 h.

**Figure 3:**
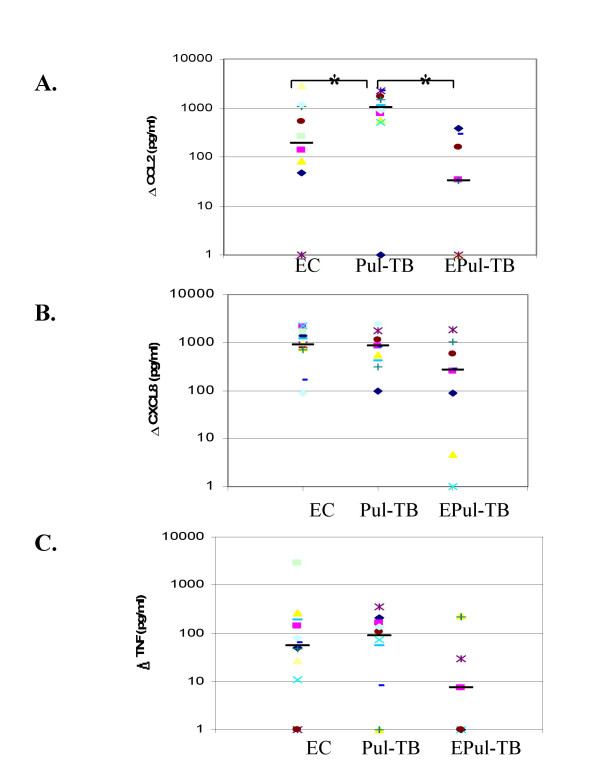
**Increased BCG-induced CCL2 secretion in pulmonary tuberculosis. **Whole blood cells were stimulated with *M. bovis *BCG as described in Fig. 2 and chemo/cytokine secretion measured at 18 h post-stimulation. Data for individual subjects; controls 'EC' (N = 12), pulmonary 'Pul-TB' (N = 10) and extra pulmonary 'EPul-TB' (N = 8) tuberculosis is illustrated. The median value is indicated by a horizontal line. Baseline levels of cytokines have been subtracted in each case 'Δ '. A. CCL2, B. CXCL8 and C. TNFα. Pair-wise differences were calculated using the Mann-Whitney U test with P < 0.05, '*'.

BCG-induced chemokine secretion in tuberculosis patients and controls at 18 h post-stimulation is illustrated in Fig. 3. Baseline levels of chemokine secretion have been subtracted and are described in the text above. BCG- induced CCL2 was significantly greater in pulmonary tuberculosis as compared with extra-pulmonary at both 18 h (P = 0.001) (Fig. 3A) and 48 h (P = 0.02, data not shown) post-stimulation. CCL2 secreted in whole blood cells from pulmonary patients was also greater as compared with controls at 18 h (P = 0.05) (Fig. 3A) and 48 h (P < 0.001, data not shown). There was no difference in BCG-induced CXCL8 between pulmonary or extra-pulmonary tuberculosis patients. Similarly, no difference was noted between patients and controls at both time points studied (18 h, Fig. 3B; 48 h, data not shown). In addition, we did not observe any differences in BCG-induced TNFα between controls and tuberculosis patients, P > 0.05 (Fig. 3C) at either time interval studied. Although the trend of TNFα secretion was lower in the extra-pulmonary group as compared with the pulmonary group, this difference was also not significant.

## Discussion

We have used an *ex vivo *whole blood assay to study differences in immune parameters in pulmonary and extra-pulmonary tuberculosis. We observed that BCG-induced CCL2 responses in pulmonary tuberculosis were greater as compared to both extra-pulmonary tuberculosis and control groups. LPS stimulated CCL2 and TNFα responses were also raised in pulmonary tuberculosis as compared with extra-pulmonary tuberculosis patients.

We included both PPD skin test positive and negative individuals to be representative of our healthy endemic population, where a large proportion of BCG vaccinated individuals remain non-reactive to the PPD skin test antigen [25]. Our tuberculosis patient group was small because of stringent selection criteria. We excluded most of those patients who had received some form of anti-tuberculous treatment for any length of time. Due to poor therapeutic practices in Pakistan the majority of patients presenting to a tertiary care center such as AKUH have received variable course anti-tuberculous therapy [26]. There were three times as many female than male patients in our study and this ratio is representative of female preponderance in the disease pattern in Pakistan [27]. In congruence with previous studies, neutrophils and monocyte counts were not significantly altered in patient groups [28]. However, erythrocyte sedimentation was significantly higher in tuberculosis patients, reflective of active infection [28]. Two extra-pulmonary tuberculosis patients (abdominal and lymphnode respectively) showed lung involvement on chest X-ray. These were grouped based on their predominant extra-pulmonary disease site and found that their responses correlated with others in the group.

The raised circulating levels of CXCL8 we observe in tuberculosis patients correlate with previous reports [16,17]. Increased CXCL8 has been associated with increased neutrophils in broncho-alveolar lavage [18] and pleural fluid [29]. However, we did not find increased neutrophils or monocytes in peripheral circulation of our patients. We observed raised TNFα in sera of tuberculosis patients. This is contrast to reports by Juffermans *et *al. who found that TNFα was not raised in sera of tuberculosis patients [30], while additional reports by Olobo *et *al. indicate that serum TNFα is increased in healthy contacts [31]. We did not observe any increase in circulating CCL2, correlating with reports by Lee *et *al. [32] who compared sera from tuberculosis patients with healthy tuberculin reactive controls from a disease endemic region. This was in contrast with Juffermans *et *al. who observed raised CCL2 in sera of tuberculosis patients as compared with healthy individuals from a non-endemic region [16].

*Ex vivo *stimulation of whole blood leucocytes provides useful information on control of cytokine secretion, since it utilizes the host environment [33]. Whole blood assays have been shown to provide useful insight into the immunopathology of tuberculosis infection [34]. The time course profiles of CCL2, CXCL8 and TNFα secretion we observed in response to BCG and LPS corresponded to previous reports of *Mycobacterium *and LPS induced activation of purified monocytes and adherent cells [19,35,36]. Further, after LPS stimulation we observed greater cytokine activation than after mycobacterial stimulation as has been shown in the case of cytokines such as CXCL8 [37]. Therefore the data we obtained using our whole blood assay correlated with patterns observed using purified subsets of cells.

Increased CCL2 in pulmonary tuberculosis may be related to localization of infection to the lung and may subsequently prevent disease dissemination. We observed raised CCL2 in pulmonary tuberculosis compared with controls in response to BCG stimulation. Raised CCL2 activation in tuberculosis correlates with previous studies [19,32,38]. However, this contrasts with reports by Lee *et *al. who did not observe any increase in CCL2 secreted by adherent cells from pulmonary tuberculosis patients as compared with healthy tuberculin reactors – in response to PPD or *M. tuberculosis *30 kD antigen [32]. One difference as compared to our study was that Lee *et *al. used healthy controls with a PPD skin test induration > 15 mm, while our control group comprised of either PPD negative or PPD positive individuals with induration > 10 mm. In addition, the use of live *Mycobacteria *and unprocessed whole blood is likely to give more physiologically relevant results for *in vivo *chemokine and cytokine activation as compared with recombinant antigen or subunit preparations. We also observed greater CCL2 activation in pulmonary tuberculosis as compared with extra-pulmonary disease in response to both BCG and LPS stimulation.

LPS-induced CXCL8 was not raised in whole blood cells from patients and this was paradoxical with increased levels in sera of these patients. Differences between *in vivo *(serum and plasma) and *in vitro *(PBMC-induced cytokine) activation have been shown previously [39]. Previously, we have shown increased spontaneous CXCL8 in disseminated mycobacterial (leprosy) infections while inducible CXCL8 was not raised in patients [40]. Therefore, increased levels of CXCL8 in sera may be indicative of pathology in these patients and may not contribute to the effective recruitment of cells.

TNFα levels were raised in pulmonary as compared with both extra-pulmonary tuberculosis and controls in response to LPS but not BCG stimulation. This difference in TNFα levels was evident at the earlier (18 h) but not later time interval. Probably, due to the reduced time course profile of TNFα by 48 h. Lack of difference in TNFα in response to BCG between groups, may also be due to reduced overall levels induced by mycobacterium as compared with the LPS.

## Conclusion

Macrophages and other polymorphonuclear cells play essential roles in initiation and maintenance of immune responses to *M. tuberculosis*. Pro-inflammatory TNFα, CCL2 and CXCL8 are important in maintaining a balance between recruitment of other macrophages and also T cells to restrict infection. CCL2 contributes to anti-mycobacterial inflammatory response by attracting monocytes and T lymphocytes. Raised CCL2 in pulmonary tuberculosis may lead to greater monocyte mediated immunity. Reduced CCL2 concomitant with lowered TNFα in extra-pulmonary tuberculosis may lead to lowered protective cellular responses and increased dissemination in these patients.

## Methods

### Subject selection and diagnosis

Patients were recruited from the Aga Khan University (AKUH) and Masoomeen Hospitals in Karachi. Ethical Clearance was obtained from the AKUH Human Subjects Protection and Ethical Review Committee and samples were taken from patients with informed consent. All study subjects were examined and investigated by infectious diseases consultants. Patients with significant co-morbid conditions including diabetes mellitus, chronic renal failure, chronic liver disease, those on high dose corticosteroid therapy and those with HIV/AIDS were excluded from this analysis. All patients were untreated except for two who had been on anti-tuberculous therapy for 2 months each due to delays in diagnostic confirmation of extra-pulmonary tuberculosis. Patients were diagnosed by clinical examination, chest X-ray, sputum *M. tuberculosis *microscopy and culture. Chest X-rays were evaluated by one of the consulting physicians using the classification of the National Tuberculosis Association of the USA into minimal, moderate and advanced lung tissue involvement [41]. Microscopy was performed using Ziehl-Neelsen staining. Cultures were done by means of a set of Lowenstein Jensen slants with and without glycerol. Purified protein derivative (PPD) skin test was carried out on each study subject. PPD skin test negative were those with induration < 10 mm, with PPD positive with induration = 10 mm. Hematological parameters for all subjects were noted on the date of enrollment and shown in Table 1. The pulmonary tuberculosis (Pul-TB) group comprised of 10 patients (3 males and 7 females) with moderate disease and no other site involved except the lung. The extra-pulmonary tuberculosis (EPul-TB) group comprised of 8 patients (2 males and 6 females) with tuberculosis in the following sites; lymph nodes (N = 3), pericardial effusion (N = 2), meningitis (N = 1), abdomen (N = 2). Of the EPul-TB patients, 6/8 were confirmed on *M. tuberculosis *smear/culture and/or radiology, while 2/8 were diagnosed on the basis of clinical presentation and a favorable response to anti-tuberculosis drugs. Two of the EPul-TB patients also showed pulmonary involvement on Chest X-ray. Twelve BCG-vaccinated healthy endemic controls (EC) (9 males and 3 females) who were staff at the AKUH were also included in the study. PPD+ volunteers had normal chest X-Ray and no clinical evidence of active disease.

### *Mycobacterium *culture

*Mycobacterium bovis *BCG (Montreal vaccine strain) was grown to logarithmic phase in 7H9 Middlebrook medium with supplements (DIFCO Laboratories, MI, USA). Mycobacteria were frozen in growth medium containing 20 % glycerol and stored at -70°C in single use aliquots for the assay as described previously [40]. Mycobacterial viability was confirmed prior to assays by fluorescent staining [42] and by colony counts on Middlebrook 7H11 agar.

### Whole blood assays

Venous blood was diluted 1: 10 in RPMI-1640 medium each well containing 10^6 ^whole blood cells was set up as per protocol [34]. Freshly thawed aliquots of BCG were washed in PBS and diluted to infect cells as described previously [43]. Each was stimulated with either *E. coli *lipopolysaccharide (LPS) (Sigma, USA) 0.1 μg/ml or mycobacteria at 2 × 10^5 ^CFU/ml (at a ratio of 0.2 bacteria per cell). Supernatants were collected at 0, 1, 6, 18, 48 and 120 h (5 days) post infection for chemokine measurement, spun to collect cellular debris and stored at -70 C until tested. BCG innoculum was tested for LPS contamination using the E-Toxate Kit (Sigma, USA).

### ELISAs

ELISA reagents for CCL2, CXCL8 and TNFα were from R&D Systems (USA). Assays were carried out according to the manufacturer's recommendation and as reported previously [43]. The lower limit of detection for CCL2 was 7.8 pg/ml, and 3.9 pg/ml for TNFα and CXCL8.

### Statistical analysis

Data is expressed as Mean (SD) or Median as described in figures. Statistical analysis was carried out using Mann-Whitney U test for Pair-wise analysis of groups using the SPSS package, USA and also ANOVA analysis.

## List of abbreviations used

BCG, bacille-Calmette Guerin vaccine strain; LPS – lipo polysaccharide; TNFα – tumor necrosis factor alpha; Pul-TB – pulmonary tuberculosis; EPul-TB – extra-pulmonary tuberculosis; EC – endemic controls; CCL2 – C-C chemokine ligand 2; CXCL8 – C-X-C chemokine ligand 8.

## Competing interests

The author(s) declare that they have no competing interests.

## Authors' contributions

This work was conceived and written up by ZH. IZ carried out the immunological assays and AK helped coordinate patient samples and *ex vivo *assays. BJ and MAK recruited patients for the study and gave input into the data analyses and interpretation. RH helped in preparation of the manuscript. All authors have read and approved the final manuscript.

## Figures and Tables

**Table 1:** Hematological characteristics of study subjects

**Group**	**Pul-TB, **N = 10 Median (range)	**EPul-TB, **N = 8 Median (range)	**Controls, **N = 12 Median (range)
**Age **(y)	25 (15 – 50)	32 (15 – 58)	26 (18–29)
**TLC **(10 xE9/L)	9.8 (7 – 22.2)	9.4 (6.4 – 16.3)	7.2 (3 – 9)
**Neutrophils **(%)	64 (38 – 87)	70 (60 – 90	56 (38.1 – 70.6)
**Monocytes **(%)	6.7 (3.6 – 9.2)	7.4 (1.7 – 11.4)	7.2 (5.8 – 9.4)
**ESR **(mm/h)	60* (40–100)	50* (20–80)	0–3
***M. tuberculosis***+^a^	9/10	3 / 8	NA
**%PPD+ **(No. tested)	80 (8)	50 (4)	50 (12)

^a ^Sputum/Biopsy/Culture positive for *M. tuberculosis*. Pulmonary tuberculosis (Pul-TB), extra-pulmonary tuberculosis (EPul-TB), purified protein derivative (PPD) skin test, total lymphocyte count (TLC), erythrocyte sedimentation rate (ESR), NA -not applicable. '*' P < 0.05 as compared with Controls.

**Table 2:** LPS-induced CCL2, CXCL8 and TNFα secretion in TB patients

**Chemo/cytokines pg/ml Median (range)**				
**Group**	**Time (h)**	**ΔCCL2**	**ΔCXCL8**	**ΔTNFα**
EC (N = 12)	18	754 (17–3058)	1712 (788–2786)	*^3 ^1153 (0–1489)
	48	1881 (713–7164)	1996 (1823–2977)	625 (211–858)
Pul (N = 10)	18	*^1 ^1218 (765–2254)	1175 (318–2770)	*^3,4 ^2154 (1244–4852)
	48	*^2 ^3398 (1643–7012)	1190 (0–3249)	957 (74–1869)
E-Pul (N = 8)	18	*^1 ^33 (0–818)	727 (0–1219)	*^4 ^577 (0–2180)
	48	*^2 ^32 (0–1194)	915 (0–1574)	73 (0–1821)

All values are 'Δ' after baseline subtraction of CCL2, CXCL8 or TNFα from each group. Pair-wise analysis was carried out to determine differences between groups using Mann-Whitney – U test '*' P ≤ 0.05. '^1–4^' defines pairs of values that were statistically significant from each other; Endemic controls (EC), Pul-TB (pulmonary tuberculosis), EPul-TB (extra-pulmonary tuberculosis).
